# The Influence of Basal Medium on Polyphenol Accumulation in Shoot Cultures of *Clerodendrum trichotomum* and *Clerodendrum colebrookianum*

**DOI:** 10.3390/molecules29245983

**Published:** 2024-12-19

**Authors:** Jan Gomulski, Marta Krzemińska, Magdalena Jochymek, Anna K. Kiss, Izabela Grzegorczyk-Karolak

**Affiliations:** 1 Department of Biology and Pharmaceutical Botany, Medical University of Lodz, 90-151 Lodz, Poland; jan.gomulski@umed.lodz.pl (J.G.); marta.krzeminska@umed.lodz.pl (M.K.); jochymek.magdalena@gmail.com (M.J.); 2 Department of Pharmaceutical Biology, Medical University of Warsaw, 02-097 Warsaw, Poland; anna.kiss@wum.edu.pl

**Keywords:** acteoside, basal growth medium, biomass accumulation, in vitro culture, phenylethanoids

## Abstract

Plants of the *Clerodendrum* genus, known for their rich phytochemical profiles, are used in traditional Chinese, Korean, Japanese, and Indian medicine to treat various ailments, including inflammation, hypertension, diabetes, hyperlipidemia, and cancer. Due to the limited natural availability of these plants, there is a growing interest in utilizing in vitro culture techniques to produce their bioactive compounds sustainably. In this study, the effects are compared of Murashige and Skoog (MS), Woody Plant medium (WP), Gamborg B5 (B5), and Schenk and Hildebrandt (SH) basal media on growth, biomass accumulation, and polyphenolic compound production in shoot cultures of *Clerodendrum colebrookianum* and *Clerodendrum trichotomum*. The composition of the culture medium significantly influenced the growth and metabolic profiles of both species. *C. trichotomum* exhibited the highest proliferation potential on WP and SH media, while *C. colebrookianum* was similar on WP, SH, and B5 media (multiplication factor of about 20). Dry weight accumulation was highest in *C. trichotomum* grown on SH medium (0.292 g/culture), while *C. colebrookianum* achieved a comparable biomass on SH and WP media (0.240 g/culture and 0.228 g/culture, respectively). The chemical analysis showed similar secondary metabolite profiles between the two *Clerodendrum* species with phenylethanoids such as acteoside being the predominant bioactive compounds in hydromethanolic extracts. WP medium was the most favorable for polyphenol accumulation in *C. colebrookianum* (64.5 mg/g DW), while the SH medium yielded the highest total polyphenol content in *C. trichotomum* (36.6 mg/g DW). In this study, the importance is underscored of basal medium selection in optimizing the in vitro production of bioactive polyphenolic compounds in *Clerodendrum* species, providing a foundation for the sustainable and scalable production of these pharmacologically significant metabolites.

## 1. Introduction

The genus *Clerodendrum* L., of the family *Lamiaceae*, includes various small trees, shrubs, and herbaceous plants. It comprises over 500 species distributed mainly in tropical and sub-tropical regions, many of which are classified as endemic [1,2]. In some countries, including China, Korea, Japan, and India, a few species have been used as folk and traditional medicines for inflammatory disorders, hypertension, diabetes, fever, malaria, dysentery, and cancer [1]. The plants are notable for their rich phytochemical profiles, including phenylethanoid glycosides, which play a key part in their pharmacological properties [3]. Two species important in Asia are *C. colebrookianum* Walp. and *C. trichotomum* Thunb. *C. colebrookianum* (syn. *C. glandulosum*), popularly known as East India glory bower, is indigenous to India, Malaysia, and Indonesia, where it is endemic [4]. *C. trichotomum* (Harlequin glory bower) is native to China, Korea, and Japan, but can be found as an ornamental plant in gardens in Europe, being relatively frost resistant among other *Clerodendrum* species [2]. In 2020, due to the decline in the extent and quality of habitats, it was put on the IUCN Red List of Threatened Species as a least-concern species [5]. The major chemical components reported in both *Clerodendrum* species include terpenoids, steroids, phenylethanoids, flavonoids, lignans, alkaloids, and anthraquinones. Phytochemical analyses have revealed that their leaves are a particularly valuable source of health-promoting phenylethanoid glycosides, especially acteoside [2,6].

Given the limited geographic distribution of *Clerodendrum* species and the challenges associated with their traditional cultivation, including the environmental dependency and inconsistent yields, commercial exploitation of the plants on a large scale may be unprofitable. Plant in vitro culture techniques offer a viable alternative for overcoming these limitations [7,8]. Advances in plant biotechnology have revolutionized the production of bioactive compounds, particularly through the use of in vitro cultivation of medicinal plants. Efficient in vitro culture systems can provide a sustainable and reliable source of medicinal compounds, circumventing the constraints of traditional cultivation methods and making production independent of geographical and climatic conditions. However, achieving high growth rates and secondary metabolite production in in vitro cultures requires the optimization of culture conditions [7,8]. A key aspect of maximizing compound yield is the optimization of the nutrient medium, typically based on defined formulations specifically designed for the establishment and maintenance of plant cultures, such as Murashige and Skoog (MS), McCown’s Woody Plant (WP), Schenk and Hildebrandt (SH), Linsmaier and Skoog (LS), or Gamborg’s B5 (B5) media [8,9,10,11,12]. Critical factors in this regard include the sources and concentrations of macro- and micronutrients, especially nitrogen and phosphorus, the presence of vitamins, and the types and concentrations of carbohydrates.

Numerous studies have demonstrated the effectiveness of shoot culture in synthesizing medically important plant metabolites [13]. However, the research on *Clerodendrum* species has predominantly focused on their pharmacological applications and phytochemical profiles, with limited attention given to the development of in vitro culture protocols [1,3,14]. Despite the traditional medicinal use and the valuable secondary metabolites produced by *Clerodendrum* species, there is a notable gap in the literature concerning the optimization of in vitro culture conditions to enhance the production of specific bioactive compounds in this genus.

In this study, the aim is to address this gap by evaluating the impact of different culture media on the growth and bioactive compound synthesis in two *Clerodendrum* species, *C. colebrookianum* and *C. trichotomum*. The primary objective was to initiate shoot cultures of these species and to compare the effects of various basal media, including MS, B5, SH, and WP (Table S1), on their proliferation, biomass accumulation, and polyphenol production. The bioactive compounds present in the obtained in vitro cultivated plant material were qualified and quantified through chromatographic analysis.

## 2. Results

### 2.1. Growth of Culture of Clerodendrum Species

To compare the effects of the medium type on the growth of *C. trichotomum* and *C. colebrookianum* shoots, the species were cultured on the following four different media: B5, MS, SH, and WP (Figures S1 and S2). The composition of the growth medium had a significant impact on growth in both species; however, no significant differences were noted in the % response for multiplication—for all treatments, most explants (94 to 100%) grew and multiplied.

**Figure 1:**
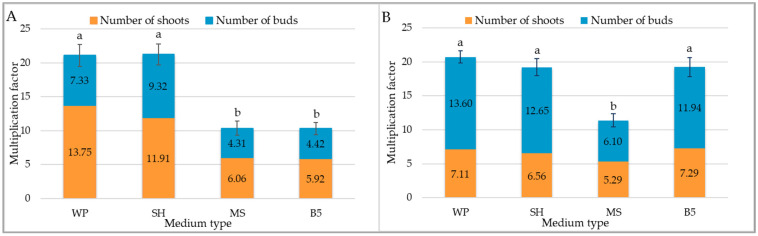
The effect of the growth media WP, SH, MS, and B5 on multiplication factor of culture of (**A**) *C. trichotomum* and (**B**) *C. colebrookianum*. The values represent the mean ± standard error of three independent experiments. Means marked with the same letter for the same species were not significantly different (*p* < 0.05).

In the case of the *C. trichotomum*, the highest mean multiplication factor, i.e., between 21 and 22, was observed when the shoots were cultivated on WP and SH medium (Figure 1A). For both of these treatments, the majority of the structures formed were shoots, although this percentage was slightly higher for WP medium than the SH medium (65% vs. 54%). The multiplication factor obtained for the culture grown on MS and B5 medium was half that observed in optimal conditions (Figure 1A).

A similar optimal multiplication factor was achieved for *C. colebrookianum* shoots grown on WP, SH, and B5 medium, i.e., 19–21 (Figure 1B). However, for this species, most of the newly formed structures were buds (54–66%), regardless of the medium used.

**Figure 2:**
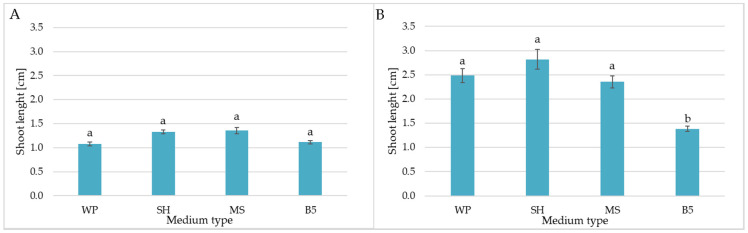
The effect of the growth media WP, SH, MS, and B5 on length of obtained shoots in culture of (**A**) *C. trichotomum* and (**B**) *C. colebrookianum*. The values represent the mean ± standard error of three independent experiments. Means marked with the same letter for the same species were not significantly different (*p* < 0.05).

Longer *C. trichotomum* shoots were obtained on the SH and MS medium, i.e., 1.3–1.4 cm; however, no statistically significant differences were found between treatments (Figure 2A). The shoots cultivated on the SH and WP media had particularly elongated internodes, while these from MS and B5 had relatively short internodes. *C. colebrookianum* shoots grown on SH, WP, and MS media were approximately twice as long as those observed for *C. trichotomum*; similar lengths were noted between these treatments (2.36–2.82 cm) (Figure 2B). Interestingly, the B5 medium clearly inhibited elongation of *C. colebrookianum* shoots.

**Figure 3:**
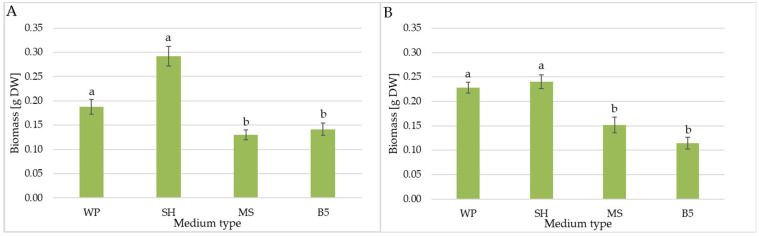
The effect of the growth media WP, SH, MS, and B5 on biomass (DW) of shoot culture of (**A**) *C. trichotomum* and (**B**) *C. colebrookianum*. The values represent the mean ± standard error of three independent experiments. Means marked with the same letter for the same species were not significantly different (*p* < 0.05).

The *C. trichotomum* cultured on SH medium obtained the highest dry weight increments (0.292 g) (Figure 3A). This was approximately a 200-fold increase compared to the inoculum. In the case of the WP medium, which is equally favorable for proliferation, the obtained dry weight value was 0.188 g, i.e., more than 30% lower. The low multiplication factor translated into relatively low increases in biomass of the culture grown on MS and B5 media. The lowest dry weight value (0.13 g) was obtained for this species on MS medium. This value was less than half that obtained in optimal conditions, on SH medium.

In the case of *C. colebrookianum*, similar dry weight values were obtained for cultures grown on SH (0.240 g) and WP (0.228 g) medium. This indicates almost an 80-fold increase in relation to the inoculum. For this species, the lowest dry weight, half that of the optimal variant, was obtained when culture was grown on B5 medium.

### 2.2. Secondary Metabolite Accumulation in Culture of Both Clerodendrum Species

**Figure 4:**
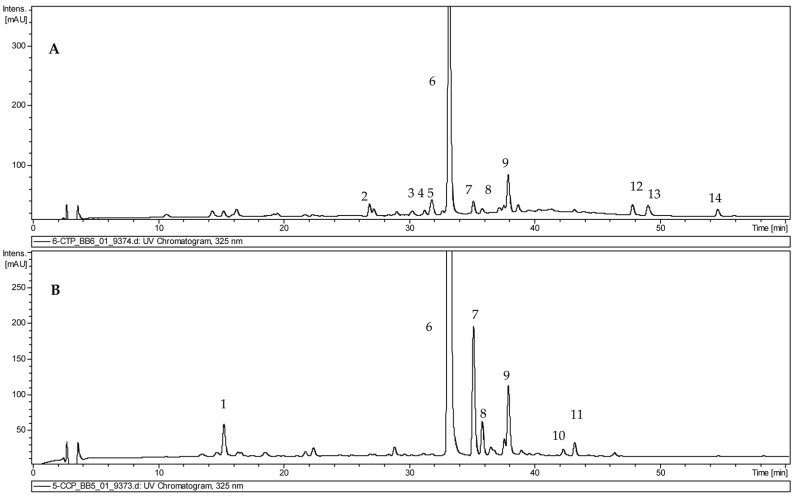
UPLC-DAD-ESI-UV chromatogram of extract (**A**) *C. trichotomum* and (**B**) *C. colebrookianum* shoot culture. Peak numbers refer to those in Table 1.

UPLC-DAD-ESI-MS analysis found the extracts from two *Clerodendrum* species to have a similar qualitative composition. The scan negative ionization mode of the *C. trichotomum* extract showed a total of eleven compounds, including eight identified as phenylethanoids (acteoside, isoacteoside, acteoside isomer, campneoside I, leucosceptoside A, *β*-hydroxyacteoside/*β*-hydroxyisoacteoside, *β*-oxoacteoside/*β*-oxoisoacteoside, hydroxylated phenylethanoid derivative), and two as flavonoids (luteolin/scutellarein hexoside derivative, apigenin); one remained unidentified (Figure 4A, Table 1). Meanwhile, in the extract of *C. colebriookianum*, five compounds were identified as phenylethanoids (acteoside, isoacteoside, acteoside isomer, leucosceptoside A, martynoside), one as a flavonoid (apigenin rhamnosyl-hexoside), and one as a phenolic acid (caffeic acid hexoside) (Figure 4B, Table 1).

Peaks **6** (Rt = 33.4 min), **7** (Rt = 35.1 min), and **8** (Rt = 35.9 min), observed in extracts from both species, exhibited a pseudomolecular [M − H]^−^ ion at *m*/*z* 623. Fragmentation of this ion gave ions at *m*/*z* 461 [M − H − 162]^−^, *m*/*z* 315 [M − H − 162 − 146]^−^, and *m*/*z* 135 [M − H − 162 − 146 − 180]^−^ corresponding to the initial cleavage of a caffeoyl residue, and then of rhamnose and hexose units. Compound **6** was identified as acteoside and **7** as isoacteoside by comparison with the reference standards and the literature data, while compound **8** was indicated as another isomer of acteoside. Acteoside and its isomers have been earlier identified in other *Clerodendrum* species [15].

Peak **9** (Rt = 37.9 min), found in both *C. colebrokianum* and *C. trichotomum*, was identified as leucosceptoside A. It has a pseudomolecular ion at *m*/*z* 637, and fragmentation ions at *m*/*z* 461 [M − H − 176]^−^, *m*/*z* 315 [M − H − 176 − 146]^−^, *m*/*z* 193, and *m*/*z* 135 [M − H − 176 − 146 − 180]^−^. The ion at *m*/*z* 461 is considered to originate from the loss of the feruloyl moiety from the parent ion at *m*/*z* 637, the ion at *m*/*z* 315 arises from the loss of the rhamnose unit from the ion at *m*/*z* 461, and the ion at *m*/*z* 135 from the further loss of hexose. The ion at *m*/*z* 193 was attributed to the presence of a feruloyl moiety; this compound has been earlier identified in *C. bungei* Steud. [16].

Peak **11** (Rt = 43.5 min) for *C. colebrookianum* gave a pseudomolecular ion at *m*/*z* 651 [M − H]^−^. The fragmentation pattern of this compound gave an ion at *m*/*z* 505 [M − H − 146]^−^ attributed to the loss of a rhamnosyl group and an ion at *m*/*z* 475 [M − H − 176]^−^ attributed to the loss of a feruloyl moiety. The ion at *m*/*z* 329 arose from the loss of the rhamnose moiety from the ion at *m*/*z* 475, and the ion at *m*/*z* 193 suggested the presence of a feruloyl moiety. The compound was identified as martynoside, isolated from other *Clerodendrum* species [16,17]. Spectral data and retention times for leucosceptoside A and martynoside were compared to those for authentic standards.

Other acteoside derivatives were present in the extract. Peak **2** (Rt = 26.9 min) for *C. trichotomum* was tentatively identified as *β*-hydroxyacteoside/*β*-hydroxyisoacteoside. It had a pseudomolecular ion at *m*/*z* 639. It demonstrated a fragmentation pattern with fragments at *m*/*z* 621 [M − H − 18]^−^ and *m*/*z* 459 [M − H − 18 − 162]^−^. The ion found at *m*/*z* 621 was consistent with water elimination, whereas the fragment at *m*/*z* 459 corresponded to the loss of the caffeic acid moiety. Meanwhile, peak **3** (Rt = 30.3 min) for *C. trichotomum* was tentatively identified as *β*-oxoacteoside/*β*-oxoisoacteoside; it gave a pseudomolecular ion at *m*/*z* 637 [M − H]^−^. The fragmentation of this compound gave an ion at *m*/*z* 475 [M − H − 162]^−^ attributed to the loss of a caffeic acid moiety, and an ion at *m*/*z* 329 [M − H − 162 − 146]^−^ attributed to the further loss of a rhamnosyl group.

Compound **4** (Rt = 31.3 min) found in the extract of *C. trichotomum* displayed a pseudomolecular ion [M − H]^−^ at *m*/*z* 639. A presence of fragments at *m*/*z* 621 [M − H − 18]^−^ suggested water elimination, and at *m*/*z* 493 [M − H − 146]^−^ loss of rhamnose. However, accurate identification was impossible, and the compound was assigned as a hydroxylated phenylethanoid derivative.

The peak **5** (Rt = 31.3 min) observed in *C. trichotomum* extract gave a pseudomolecular ion at *m*/*z* 653 [M − H]^−^ which was tentatively identified as campneoside I, previously isolated from *C. inerme* (L.) Gaertn. [18]. It gave a fragment at *m*/*z* 621 which represented the loss of a methoxyl group, and an ion at *m*/*z* 459 corresponding to the further loss of the caffeoyl moiety.

Peaks **10** found in extract of *C. colebrookianum*, and **12** and **14** in *C. trichotomum* were defined as flavonoids. Compound **12** (Rt = 48.1 min) was identified as luteolin/scutellarein hexoside. It has a pseudomolecular ion at *m*/*z* 571, and a fragmentation ion at *m*/*z* 285, corresponding to luteolin/scutellarein aglycon resulting from a loss of hexose unit [M − H − 162]^−^ from a fragmentation ion at *m*/*z* 447 [M − H]^−^. Peak **14** (Rt = 54.7 min) exhibited a pseudomolecular ion at *m*/*z* 269 was identified as apigenin by comparison with reference standards. Peak **10** (Rt = 42.3 min) exhibited a pseudomolecular ion at *m*/*z* 577; its fragmentation gave ions at *m*/*z* 431 [M − H − 146]^−^ and *m*/*z* 415 [M − H − 162]^−^, corresponding to the loss of rhamnose and hexose units, and at *m*/*z* 269 [M − H − 146 − 162]^−^ corresponding to the apigenin aglycon resulting from the loss of rhamnose and hexose units.

Moreover, peak **1** (Rt = 15.4 min) found in the *C. colebrookianum* extract exhibited a pseudomolecular [M − H]^−^ ion at *m*/*z* 341, and fragmentation ion at *m*/*z* 179 due to the loss of a hexoside residue, which suggests the presence of a caffeic acid hexoside.

Peak **13** (Rt = 49.2 min), from the *C. trichotomum* extract displaying pseudomolecular ion [M − H]^−^ at *m*/*z* 563 and fragments at *m*/*z* 388, remained unidentified.

**Figure 5:**
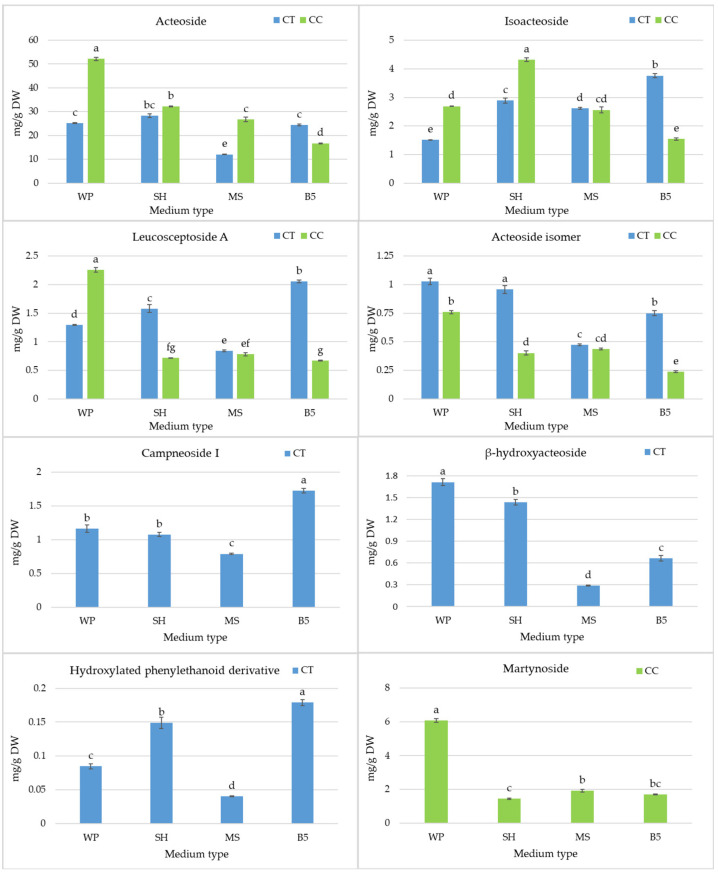
The effect of the growth media WP, SH, MS, and B5 on phenylethanoid accumulation in shoot culture of *C. trichotomum* (CT) and *C. colebrookianum* (CC). The values represent the mean ± standard error of three independent experiments. Means marked with the same letter for the same metabolite were not significantly different (*p* < 0.05).

**Figure 6:**
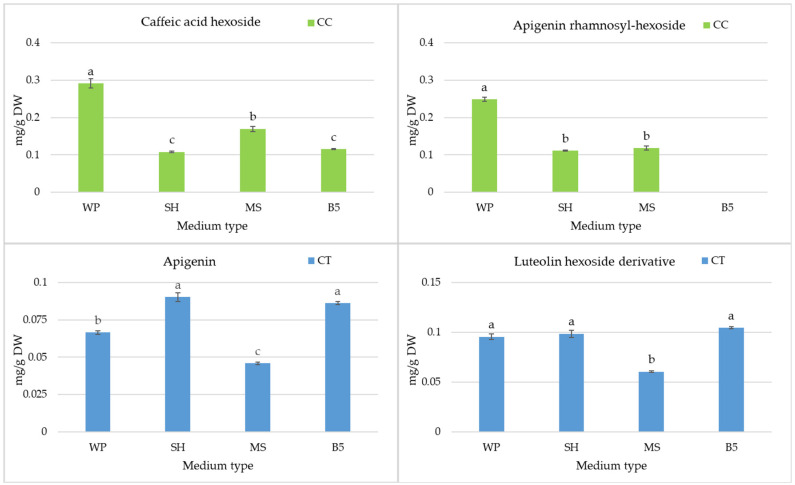
The effect of the growth media WP, SH, MS, and B5 on other metabolite accumulation in shoot culture of *C. trichotomum* (CT) and *C. colebrookianum* (CC). The values represent the mean ± standard error of three independent experiments. Means marked with the same letter for the same metabolite were not significantly different (*p* < 0.05).

It was possible to acquire significantly increased levels of secondary metabolites in *Clerodendrum* culture via optimization of the basal medium. For both species, acteoside accounted for approximately 80% of the summed content of all polyphenols in the extracts. However, its level was higher in the optimal conditions in *C. colebriookianum* shoots than in *C. trichotomum*. The highest acteoside content in *C. colebriookianum* was obtained in the culture growing in WP medium (52.14 mg/g DW) (Figure 5). Changing the culture medium resulted in a drastic decrease in acteoside in shoots, and the least favorable medium, B5, reduced its level by more than three times. Also, all other metabolites detected in the *C. colebrookainum* culture, except isoacteoside, reached their maximum during growth on WP medium (Figure 5 and Figure 6). The highest content of the second most abundant metabolite, isoacteoside, was observed in the shoots of *C. colebrookianum* on SH medium (4.32 mg/g DW).

*C. trichotomum* shoots accumulated similar levels of acteoside during cultivation on WP, SH, and B5 media (between 24.4–28.3 mg/g DW) (Figure 5), while a considerable fall in acteoside content was noted for MS medium. SH and WP media also obtained the highest levels of the acteoside derivative in the *C. trichotomum* culture (Figure 5). However, the highest levels of most phenylethanoids (isoacteoside, leucosceptoside A, campneoside I, and hydroxylated phenylethanoid derivative) were obtained in those shoots cultivated on B5 medium. In this culture, *β*-oxoacteoside was present only in trace amounts, regardless of the basal medium. The highest levels of flavonoids were found in *C. trichotomum* shoots during their growth in SH and B5 medium (Figure 6).

**Figure 7:**
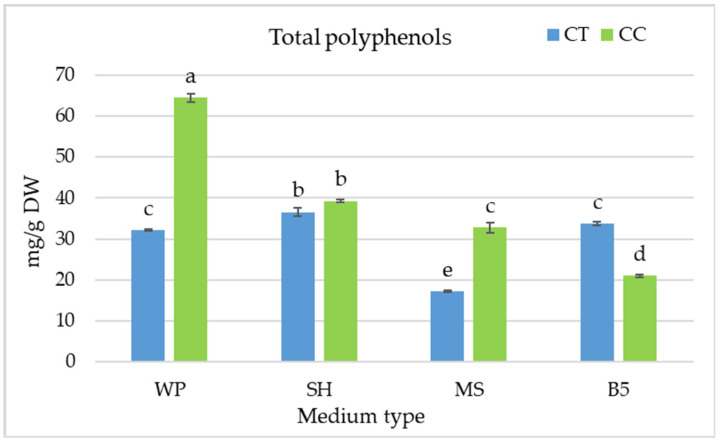
The effect of the growth media WP, SH, MS, and B5 on the total polyphenol content in shoot culture of *C. trichotomum* (CT) and *C. colebrookianum* (CC). The values represent the mean ± standard error of three independent experiments. Means marked with the same letter were not significantly different (*p* < 0.05).

Ultimately, the greatest production of bioactive compounds in *C. colebriookianum* shoots was noted on WP medium, which yielded 64.5 mg/g of total polyphenols (Figure 7). In contrast, in the case of *C. trichotomum*, the SH medium turned out to be the most favorable (36.6 mg/g DW); however, this value was only 7–10% higher than on WP and B5 medium (32.2–33.8 mg/g DW).

## 3. Discussion

With a promising medicinal potential and the ornamental value, limited access to *Clerodendrum* species due to their often endemic nature encourage the search for alternative methods of obtaining large amounts of plant material in a relatively short time and for obtaining consistently high levels of production of medicinal compounds. Such perspectives can be provided by plant in vitro culture. However, for the genus *Clerodendrum*, relatively few of the published studies are based on biotechnological tools. No literature data on the use of tissue cultures for obtaining *C. trichotomum* currently exist, and only one report has described the culture of *C. colebrookianum*. Previous research of in vitro *Clerodendrum* spp. culture has focused on the development of micropropagation protocols, rather than secondary metabolite production, and included only a few species. All were carried out on a solid medium, agar was most often chosen as the gelling substance, and the carbon source for all the above cultures was sucrose [14].

An important factor influencing the growth of the culture and the production of secondary metabolites, is the growth medium [8]. Although no data exist regarding the optimization of the growth medium for *Clerodendrum* spp., such experiments have been performed for other species [19,20,21]. In the present study, the most effective medium for growth and proliferation was found to be WP for *C. colebrookianum*, and SH, followed by WP for *C. trichotomum*. SH medium was not included in any of the previous reports on *Clerodendrum* species.

Most earlier experiments on plants from the *Clerodendrum* genus were based on MS medium [22,23,24,25], the standard medium used for shoot cultures [26], although two studies examined the effects of WP medium [27,28]; however, neither analyzed the effect of medium type on proliferation efficiency. While a nodal explant of *C. serratum* (L.) Moon has been used for experiments on MS and WP, with the same combination of BAP, these were derived from two independent studies [23,28]; the findings indicated that *C. serratum* yielded a mean of 2.5 shoots per explant cultivated on MS, and 4.5 shoots on WP medium. This indicates, as in our experiment, that the WP medium was more effective than the MS medium in the multiplication of *C. serratum*. Additionally, compared with our present data, it seems that *C. serratum* has a weaker potential for clonal multiplication than *C. trichotomum* or *C. colebrookianum*.

The efficiency of clonal multiplication reported for *Clerodendrum* species varies greatly, ranging from 2 for *C. phlomidis* L. f. to 20 for *C. colebrookianum* [14]. For *C. phlomidis* nodes cultivated on MS medium, shoot multiplication ranged from only 1 to 2.3, depending on the added cytokinin; this was due to the strong callus formation on explants [29]. However, Devika and Kovilpillai [24] obtained eight shoots per cultured explant from the same species on MS medium. A similar proliferation factor was observed for *C. viscosum* Vent. [25], while half this was noted under optimal conditions for *C. incisum* (L.) Kuntze (four shoots/node) [30] and *C. inerme* (three shoots/node) [31]. The highest multiplication factor among those described for the genus, and similar to that in our study, was obtained for nodal explants of *C. colebrookianum* [27]. However, this level of regeneration was obtained by supplementing the WP medium with twice the BAP (6-benzylaminopurine) content than in our present experiment; the use of BAP at a concentration of 0.5 mg/L reduced the number of *C. colebrookianum* shoots formed almost threefold [27]. The second species analyzed herein, *C. trichotomum*, also demonstrated exceptionally high regeneration potential compared to previous studies of *Clerodendrum*.

Moreover, despite some differences, both tested species favor growth and proliferation media with poorer micro- and macroelement content than MS medium [9,11,12]. The differences in the response to the basal medium indicate differences in the demand for nutrients. WP medium has a much lower ionic strength than MS, with several times lower levels of nitrogen, and no cobalt or iodine ions at all. In contrast, the most favorable medium for the growth of *C. trichotomum*, SH medium, is characterized by lower nitrogen levels than MS and an exceptionally low ratio of ammonium to nitrate ions. Moreover, with a significantly lower content of Ca, Mn, Zn, and Mo ions, it has higher levels of other components such as Co and Cu ions and many vitamins.

Studies on various plant species have found the choice of growth medium may have a decisive influence on the production of secondary metabolites in vitro [8]. However, no *Clerodendrum* shoot cultures have been analyzed for their bioactive compound content. Only two studies have analyzed the production of secondary metabolites in cultures of *Clerodendrum* species [24,32], and no attempts have been made to optimize the biosynthesis of the compounds by modifying culture conditions. In the first, collected and dried leaves of *C. phlomoides* were subjected to extraction with various solvents, with the initial analyses confirming the presence of steroids, polyphenols, and alkaloids without their identification in the individual extracts [24]. In the second, a protein conferring resistance against viruses was isolated from the aqueous extract of *C. acuelatum* (L.) Schltdl. shoot culture [32].

The use of UPLC-DAD-ESI-MS analysis allowed us to assess for the first time the polyphenol profile of in vitro cultures of two *Clerodendrum* species, and it indicated that they are able to produce compounds typical for the parent plant. Phenylethanoids, the main group found in both species, are crucial for the composition and activity of these plants growing in natural conditions. They have been documented to be useful in many diseases and have demonstrated various activities including neuroprotective, anti-inflammatory, antioxidant, anti-aging, neuroprotective, memory enhancement, antibacterial, antivirus, cytotoxic, immunomodulatory, antidiabetic, and anti-obesity [33,34].

The shoot cultures of *C. trichotomum* and *C. colebrookianum* showed similar qualitative profiles of polyphenol. Noteworthy are the greater diversity of secondary metabolites in *C. trichotomum* shoots, while higher levels were noted in *C. colebrookianum* culture. Among the phenylethanoids, hydroxy- and oxo-derivatives of acteoside, campneoside I, and one more compound from this group were identified in the *C. trichotomum* extract for the first time. The UPLC-DAD-ESI-MS analyses detected the following two flavonoids in *C. trichotomum* culture: apigenin and luteolin derivative/scutellarein. While apigenin and its derivatives had been found before in *C. trichotomum* leaves and flowers [35], neither luteolin nor scutellarein derivatives were found in the extracts from parent plants. However, derivatives of both of these flavonoids were identified from the mother plants of *C. colebrookianum*. Few reports exist on the chemical composition of this species and most analyses are based on the estimation of the total polyphenol and/or flavonoid content in the studied extract. The phytochemical profile of the water–ethanol extract of the roots and leaves of the parent plant was presented by Deb et al. only in 2021 and 2022 [36]; while these reports describe most of the phenylethanoids identified herein, as well as caffeic acid, our present findings are the first to mention the presence of leucosceptoside A in this species, as well as another derivative of apigenin than previous detected.

For both *Clerodendrum* shoot cultures, the applied conditions had a significant influence on the individual and total yields of the detected polyphenols. In the case of *C. trichotomum*, a favorable accumulation of predominated acteoside was noted during shoot growth on SH medium (28.3 mg/g DW). SH medium also yielded a higher content of some other metabolites in the *C. trichotomum* culture, although some preferred the B5 medium. In contrast, extremely high amounts of acteoside were found in *C. colebrookianum* WP culture; these levels were three-fold higher than in the shoot biomass from the B5 medium, and two times higher than for optimal conditions in *C. trichotomum*. WP provided the most favorable conditions for the biosynthesis of most compounds of this species. This medium was also optimal for the production of polyphenols in *Gardenia jasminoides* culture; meanwhile, some of the secondary metabolites of gardenia were produced only in trace amounts during cultivation on MS medium [37]. MS was also found to be the least beneficial medium for biosynthesis in our present study. Similarly, the growth of *Clidemia hirta* L. shoots resulted in a significantly higher accumulation of saponins during the culture on WP medium compared to the treatment with MS medium [19]. However, SH medium proved to be the most beneficial for bioactive compound production in the culture of *Salvia bulleyana* Diels. [38], and the B5 medium in shoots of *Moringa oleifera* (L.) Small [39].

Most studies to date on *Clerodendrum* spp. have almost exclusively used the original MS medium [22,23,24,25]. Our study is the first to examine the effects of different media on secondary compound production for these species. As a result, the standard MS medium was not found beneficial for the high yield production of phenylethanoids in either *Clerodendrum* culture. Compared to MS medium, two-fold increases in total polyphenol content were achieved during cultivation in optimal conditions, i.e., on WP medium for *C. colebrookianum*, and SH for *C. trichotomum*. The highest polyphenol content was characterized by media with significantly lower nitrogen concentrations than in MS [9,10,11,12]. The NO_3_^−^:NH_4_^+^ ratio in growth media also had an effect on biosynthesis in these cultures, and this reaction differed between the two *Clerodendrum* species. SH medium was particularly advantageous for metabolite biosynthesis in *C. trichotomum* which is characterized by a higher level of total nitrogen, but a lower NH_4_^+^/NO_3_^−^ ratio than WP medium, which was optimal for the bioactive compound production in *C. colebrookianum*.

Numerous previous studies have found that the total nitrogen content and its source, and the nitrogen/carbon ratio in the medium can significantly modify plant metabolism [40]. The nitrogen concentration and NH_4_^+^/NO_3_^−^ ratio as its sources had significant effects on individual phenylethanoid biosynthesis in *Plantago lanceolata* L. culture [41]. The conventional MS medium with 60 mM nitrogen was one of the worst for production in the culture, while the highest acteoside content was observed in the culture grown in medium containing six times lower N content (10 mM). Some researchers attribute the low biosynthesis response on MS medium to its high total nitrogen or high ammonium concentration: indeed, media with lower total nitrogen and lower NH_4_^+^ content than MS were found to be better for secondary metabolite production in *Salvia vidis* L. roots and *Echinacea angustifolia* DC. shoots [42,43]. Also, cultivation on media with lower total nitrogen and NH_4_^+^ concentrations than in MS medium resulted in most effective regeneration for a number of *Citrus* cultivars [21]. It has been hypothesized that such increases observed during nitrogen deficiency may be due to changes in the carbon/nitrogen balance; with limited nitrogen availability in plant tissues and the resulting stress, the excess carbon is driven from the pool intended for growth towards the synthesis of stress response metabolites, such as polyphenol [44].

It is not possible to compare the productivity of the *Clerodendrum* shoot cultures obtained herein to others obtained for other plants of this genus, because no such reports exist. Only one study has examined the content of acteoside in the callus of various plants including *C. trichotomum*, and its level in this material (11.8 mg/g DW) was half that of our optimized shoot culture [45]. However, acteoside has been detected in many other species from the families Lamiaceae, Plantaginaceae, and Scrophulariaceae and it is known that it can be efficiently biosynthesized in vitro. In vitro, the source of acteoside was the root cultures [46], hairy roots [47,48,49], and shoot cultures [50,51,52]. Interestingly, the production of this phenylethanoid also occurred in undifferentiated cell and suspension cultures [50,51,53,54,55]. Plant in vitro cultures were often not only stable, but also an efficient source of acteoside, with yields ranging from several milligrams to over a hundred milligrams per 1 g of dry biomass. Most often, however, the level of acteoside ranged from 10 to 25 mg/g DW [46,47,48,49,50,51,53]; therefore, the cultures of two *Clerodendrum* species described in this paper, especially *C. colebrookianum*, seem very promising and encourage further manipulations aimed at stimulating the biosynthesis of this compound. Moreover, a simple and cheap modification of the medium significantly increased the phenylethanoid yields in *Clerodendrum* cultures, and such changes could be easily implemented in industrial-scale cultures.

## 4. Materials and Methods

### 4.1. Plant Material, Culture Establishment, and Culture Conditions

In this study, shoot cultures of two *Clerodendrum* species obtained from seeds were used. The seeds of *C. trichotomum* var. *fargesii* (Dode) Rehder were obtained from the Botanical Garden Teplice (Teplice, Czech Republic) (50.6383561 N, 13.8418069 E), and *C. colebrookianum* Walp. from the Botanical Garden of the University of Zurich (Switzerland) (47.3585047 N, 8.5605700 E). The seeds of both species were carefully peeled from seed coats and rinsed with ethanol. They were then placed in a 5% sodium hypochlorite solution for five minutes. After this time, the seeds were rinsed three times with sterile distilled water and placed on MS agar medium and incubated in the dark. The seeds germinated after about 10 days. After two weeks, the apical part and nodes of the shoots developed from seedlings were transferred to MS medium with 0.1 mg/L IAA (indole-3-acetic acid) and 0.5 mg/L BAP (6-benzylaminopurine) and used to obtain shoot culture.

The explants in the experiment were stem fragments of these in vitro grown shoots, approximately 2–5 mm long, containing a single node (9–12 nodes per one repetition). The mean fresh weight of the explant of *C. trichotomum* was 11.7 mg ± 1.0 mg and dry weight was 1.45 mg ± 0.12; the respective biomasses of the inoculum of *C. colebrookianum* were 19.8 mg ± 2.2 and 3.1 mg ± 0.3. Three explants were placed in 300 mL Erlenmeyer flasks containing 70 mL of medium culture. Culture was carried out on four types of defined media. These were MS, WP, SH, and B5 medium. All variants of the medium were supplemented with the same combination of growth regulators, as follows: 0.1 mg/L IAA and 0.5 mg/L BAP. The basal media and growth regulators were purchased from Duchefa Biochemie (Haarlem, The Netherlands). Before sterilization, the media were adjusted to a pH in the range of 5.6–5.9 and solidified with agar in the amount of 7.5 g/L. The first subculture achieved after transfer to different base media was treated as adaptive and its results were not included in the analyses. Cultures were carried out in a growth chamber under a photoperiod (16 h light/8 h dark) under a light intensity of 65 µM/m^2^s, at a temperature of 26 ± 2 °C, and a relative humidity of 80–90%. The material was subcultured every six weeks.

### 4.2. Criteria for Assessing the Growth of Culture

After the subculture period, % of explants forming buds and shoots, the number of shoots and buds, the length of obtained shoots, and the dry weight (DW) of the culture expressed in grams were determined; structures less than 0.5 cm in length were considered as buds. Additionally, the multiplication factor expressing the mean number of shoots and buds produced on a single explant were calculated.

### 4.3. Phytochemical Analysis

Plant material from shoots of the two *Clerodendrum* species cultivated on the studied media were extracted and analyzed for the production of bioactive metabolites. Solvents used for extraction and phytochemical analyses were purchased from Sigma-Aldrich (St. Louis, MO, USA).

#### 4.3.1. Extraction Procedure

The freeze-dried plant material from each treatment for the two *Clerodendrum* species was ground in a mortar. Powdered samples weighing 100 mg were extracted as follows: firstly, the cultures were treated three times with 15 mL chloroform for 15 min with ultrasonification (Techpan, Warsaw, Poland). After removing most of the chlorophyll, the dry plant material was extracted once with pure methanol and then twice with a mixture of methanol: water in a ratio of 8:2, each time using 10 mL of solvent. Extraction was carried out in an ultrasonic bath for 15 min at 40 °C. The fractions were combined and evaporated to dryness under reduced pressure.

#### 4.3.2. Qualitative Analysis

The obtained extracts were dissolved in 0.1% formic acid-MeOH (8:2) and then filtered through a 0.45 μm Chromafil^™^ membrane (Macherey-Nagel, Duren, Germany). UPLC-DAD-ESI-MS analysis was performed using an UPLC-3000 RS system (Dionex, Germering, Germany) with DAD detection and an AmaZon SL ion trap mass spectrometer with ESI interface (Bruker Daltonik GmbH, Bremen, Germany). Separation was performed on a Zorbax SB C18 column (150 × 2.1 mm, 1.9 μm) (Agilent, Santa Clara, CA, USA). The mobile phase consisted of 0.1% formic acid in water (A) and 0.1% formic acid in acetonitrile (B), using the following gradient: 0–60 min, 5–40% B. The parameters of the MS unit were as follows: nebulizer pressure 40 psi, drying gas flow rate 9 L/min, nitrogen gas temperature 300 °C, and capillary voltage 4.5 kV. The detailed conditions of the analysis were as described previously [56]. The mass spectra were registered by scanning from *m*/*z* 100 to 2200. UV spectra were recorded over a range of 200–450 nm and chromatograms were acquired at 325 nm. The compounds were analyzed in negative ion mode, and identified by comparing the data on their retention time, UV–vis, and mass spectra with the data obtained for standard compounds, or the data reported in the literature [57,58,59].

#### 4.3.3. Quantitative Analysis

Before chromatographic analysis, dry extracts were dissolved in 80% methanol of HPLC analysis grade and filtered through a membrane filter with a pore diameter of 0.22 µm into HPLC vials. The analysis was performed using an Agilent Technologies 1290 Infinity liquid chromatography instrument (Agilent Technologies, Santa Clara, CA, USA). The separation was performed on an InfinityLab Poroshell120 EC-C18 column (Agilent Technologies, Santa Clara, CA, USA) (4.6 × 150 mm, 4 µm). The injection volume was set to 10 μL. Two phases were used in the analysis as follows: A, which was acetonitrile, and B, which was 0.1% formic acid. The distribution was carried out according to the following scheme: 0–15 min 10% A, 15–35 min 10–28% A, 35–37 min 28–100% A, 37–40 min 100% A. Post-time was set at five minutes. The analysis was performed at 35 °C and the flow rate was 1.8 mL/min. The analysis was monitored at λ = 330 nm. The content of secondary metabolites was determined by comparing its peak area in the analyzed samples with the data for the previously obtained calibration curve. Calibration was performed using the standard acteoside, isoacteoside, leucosceptoside, and martynoside, purchased from Chem Faces Biochemical Co., Ltd. (Wuhan, China), and caffeic acid, apigenin, and its glucoside from Sigma Aldrich (Darmstadt, Germany).

For every compound, a calibration curve was obtained by plotting the peak areas versus the content of standards in the concentration range, as follows: 50–2000 µg/mL for acteoside, and 50–1000 µg/mL for other compounds. Seven-point calibration equation was established as follows: for acteoside y = 7.21071407x (R^2^ = 0.999), and five-point for isoacteoside y = 4.88729674x (R^2^ = 0.999), for leucosceptoside A y = 3.79160256x (R^2^ = 0.999), for martynoside y = 3.64121781x (R^2^ = 0.999), caffeic acid y = 20.3905046x (R^2^ = 0.993), apigenin y = 22.0260672x (R^2^ = 0.999), and apigenin glucoside y = 11.4590443x (R^2^ = 0.999). When a pure reference standard was not available, the levels of tentatively identified compounds were expressed as the equivalents of similar standards; *β*-hydroxyacteoside, isomer of acteoside, and hydroxylated phenylethanoid derivative as acteoside; apigenin rhamnosyl-hexoside and luteolin hexoside derivative as apigenin glucoside, caffeic acid hexose as caffeic acid, and campneoside I as martynoside.

The metabolite level was expressed as mg/g dry weight (DW) of plant material. The total content of all quantified polyphenols in the samples was summed to calculate the total phenolic content.

#### 4.3.4. Statistical Analysis

All results are the mean values of three passage repetitions ± standard error (SE). Means, standard deviations, and standard errors were calculated with Excel 2013 (Microsoft Inc., Redmond, WA, USA) Statistical analysis was carried out using STATISTICA 13.1 (StatsoftPolska, Kraków, Poland). The data were compared using analysis of variance (ANOVA) followed by Tukey’s post hoc test. Differences were considered significant at a level of 5%. Results that do not differ significantly are marked on the figures with the same letters of the alphabet.

## 5. Conclusions

In this study, the effect is shown of four defined growth media (MS, WP, B5, and SH) on the growth of *C. trichotomum* and *C colebrookianum* shoots and on the production of secondary metabolites in these cultures. UPLC-DAD-ESI-MS analysis showed that the profile of polyphenols in shoot cultures is similar to that found for the aerial parts of the parent plants. Both cultures proved to be good sources of phenylethanoids, especially the dominant acteoside, with *C. colebrookianum* accumulating almost twice as much of this compound as *C. trichotomum*. The *C. colebrookianum* shoots demonstrated optimal growth and production on WP medium. In contrast, *C. trichotomum* achieved higher biomass on SH medium, which also provided optimal conditions for the production of the dominant compound, acteoside, and total polyphenol content. The limited phytochemical studies, the scarcity of biotechnological research on bioactive compounds in *Clerodendrum* species, and promising results make this work relevant, innovative, and support the use of these plants in pharmaceutical applications. Further research should aim to optimize other culture conditions for improving metabolite biosynthesis and increase the scale of cultivation.

## Figures and Tables

**Table 1:** UPLC-DAD-ESI-MS data of compounds detected in extract of *C. trichotomum* (CT) and *C. colebrookianum* (CC) shoot culture.

Peak No	Rt	[M − H]^−^	Main Fragments	Tentative Compound	*Clerodendrum* Species
**1**	15.4	341	281, 203, 179, 161, 135	caffeic acid hexoside	CC
**2**	26.9	639	621, 529, 459, 351	*β*-hydroxyacteoside/*β*-hydroxyisoacteoside	CT
**3**	30.3	637	475, 329	*β*-oxoacteoside/*β*-oxoisoacteoside	CT
**4**	31.3	639	621, 529, 493, 461	hydroxylated phenylethanoid derivative	CT
**5**	31.9	653	621, 459	campneoside I	CT
**6**	33.4	623	461, 315, 135	acteoside *	CC, CT
**7**	35.1	623	461, 315, 135	isoacteoside *	CC, CT
**8**	35.9	623	461, 315, 135	acteoside isomer	CC, CT
**9**	37.9	637	491, 461 315, 193, 135	leucosceptoside A *	CC, CT
**10**	42.3	577	415, 431, 269	apigenin rhamnosyl-hexoside	CC
**11**	43.5	651	505, 475, 329, 193	martynoside *	CC
**12**	48.1	571	447, 285	luteolin/scutellarein hexoside derivative	CT
**13**	49.2	563	388	unidentified	CT
**14**	54.7	269	-	apigenin *	CT

* compound identified after comparison with standard compound.

## Data Availability

The original contributions presented in this study are included in the article/Supplementary Materials. Further inquiries can be directed to the corresponding author.

## Supplementary Materials

The following supporting information can be downloaded at: https://www.mdpi.com/article/10.3390/molecules29245983/s1, Table S1: composition of basal media used in this experiment; Figure S1: *Clerodendrum trichotomum* shoot culture; Figure S2: *Clerodendrum colebrookianum* shoot culture.
