# Supplementary material for: The Influence of Basal Medium on Polyphenol Accumulation in Shoot Cultures of Clerodendrum trichotomum and Clerodendrum colebrookianum
Source: Molecules. 2024 Dec 19;29(24):5983. doi: 10.3390/molecules29245983 (PMC11677968; doi:10.3390/molecules29245983)

Table S1. Composition of basal media used in the experiment.

Basal medium	WP	SH	MS	B5
Micronutrient components (mg/L)				
$CoCl_2·6H2O$	-	0.10	0.025	0.025
$CuSO_4·5H_2O$	0.25	0.20	0.025	0.025
FeNaEDTA	36.70	19.80	36.70	36.70
H_3BO_3	6.20	5.00	6.20	3.00
KI	-	1.00	0.83	0.75
$MnSO_4·H_2O$	22.30	10.00	16.90	10.00
$Na_2MoO_4·2H_2O$	0.25	0.10	0.25	0.25
$ZnSO_4·7H_2O$	8.60	1.00	8.60	2.00
Macronutrient components (mg/L)				
$CaCl_2$	72.50	151.00	332.02	113.23
KH_2PO_4	170.00	-	170.00	-
KNO_3	-	2500.00	1900.00	2500.00
$MgSO_4$	180.54	195.05	180.54	121.56
NH_4NO_3	400.00	-	1650.00	-
NaH_2PO_4	-	-	-	130.44
$(NH_4)_2SO_4$	-	-	-	134.00
$Ca(NO_3)_2·4H_2O$	471.26	-	-	-
K_2SO_4	990.00	-	-	-
$(NH_4)H_2PO_4$	-	300.00	-	-
Vitamins (mg/L)				
Glycine	2.00	-	2.00	-
myo-Inositol	100.00	1000.00	100.00	100.00
Nicotinic acid	0.50	5.00	0.50	1.00
Pyridoxine HCl	0.50	0.50	0.50	1.00
Thiamine HCl	1.00	5.00	0.10	10.00
Sucrose (g/L)	30.0	30.0	30.0	30.0

Figure S1. *Clerodendrum trichotomum* shoot culture. Inoculum in the Erlenmeyer flask (A); 6-week-old culture from: WP medium (B), SH medium (C), B5 medium (D), MS medium (E) (subculture 24).

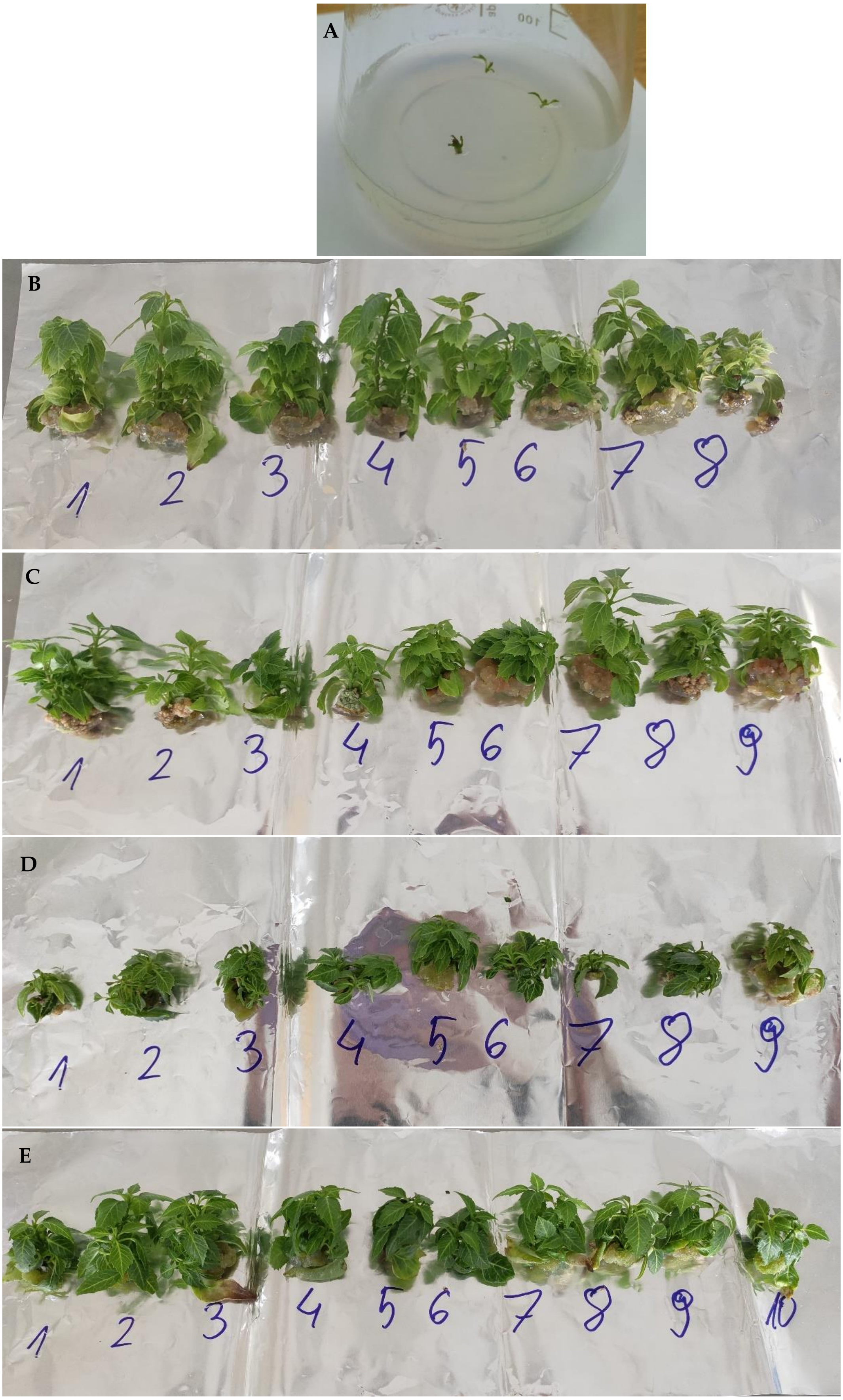

Figure S2. *Clerodendrum colebrookianum* shoot culture. Inoculum in the Erlenmeyer flask (A); 6-week-old culture from: WP medium (B), SH medium (C), B5 medium (D), MS medium (E) (subculture 24).

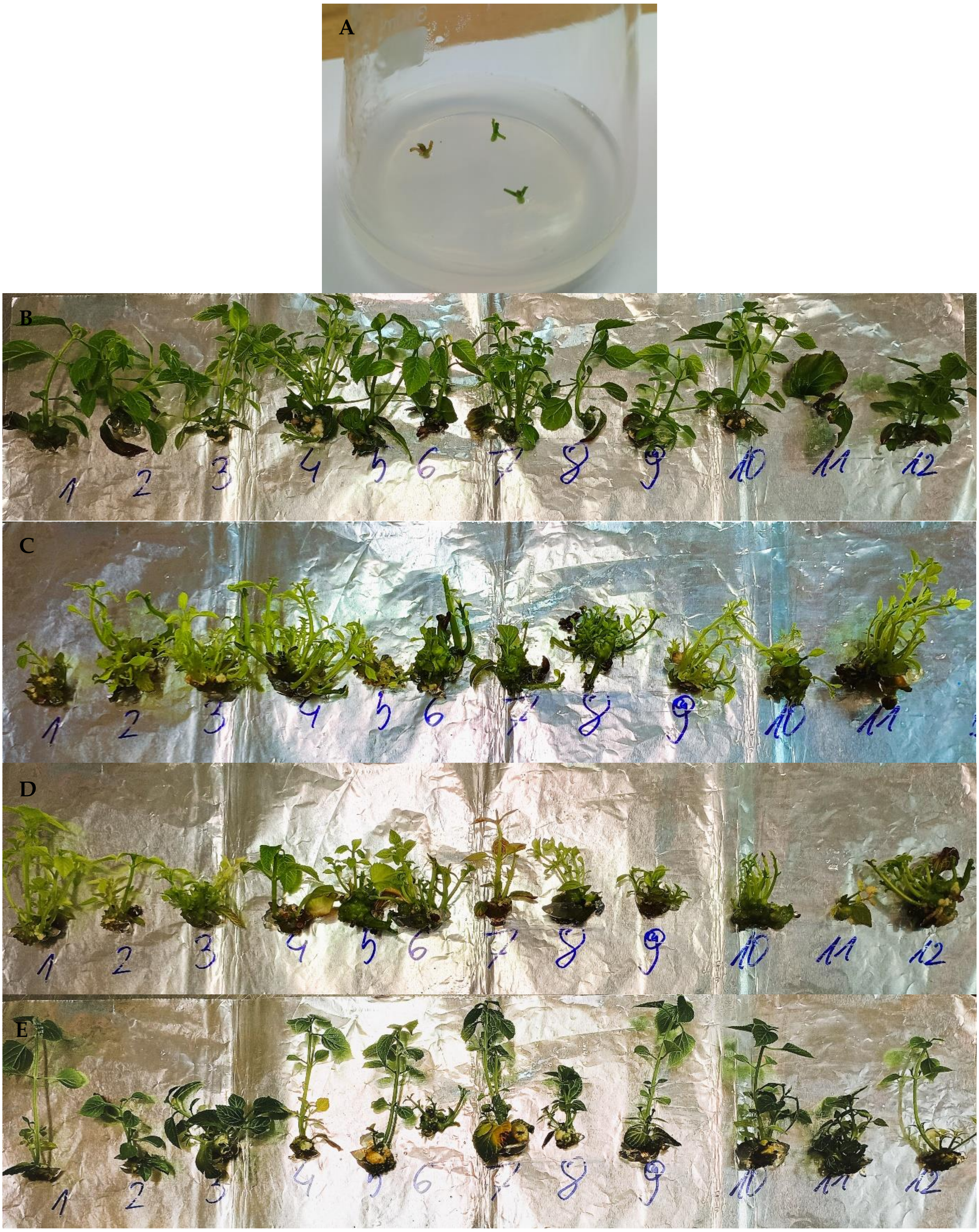

Supplement: Supplementary file 1 [file molecules-29-05983-s001.zip › molecules-3317602-supplementary.pdf]
